# Push-Broom-Type Very High-Resolution Satellite Sensor Data Correction Using Combined Wavelet-Fourier and Multiscale Non-Local Means Filtering

**DOI:** 10.3390/s150922826

**Published:** 2015-09-10

**Authors:** Wonseok Kang, Soohwan Yu, Doochun Seo, Jaeheon Jeong, Joonki Paik

**Affiliations:** 1 Department of Image, Chung-Ang University, 84 Heukseok-ro, Dongjak-gu, Seoul 06974, Korea; E-Mails: kandws12@cau.ac.kr (W.K.); shyu@cau.ac.kr (S.Y.); 2 Department of Satellite Data Cal/Val Team, Korea Aerospace Research Institute, 115 Gwahangbo, Yusung-Gu, Daejeon 34133, Korea; E-Mails: dcivil@kari.re.kr (D.S.); jjh583@kari.re.kr (J.J.)

**Keywords:** destriping, denoising, satellite data correction, combined wavelet-Fourier filter, non-local means (NLM) filter

## Abstract

In very high-resolution (VHR) push-broom-type satellite sensor data, both destriping and denoising methods have become chronic problems and attracted major research advances in the remote sensing fields. Since the estimation of the original image from a noisy input is an ill-posed problem, a simple noise removal algorithm cannot preserve the radiometric integrity of satellite data. To solve these problems, we present a novel method to correct VHR data acquired by a push-broom-type sensor by combining wavelet-Fourier and multiscale non-local means (NLM) filters. After the wavelet-Fourier filter separates the stripe noise from the mixed noise in the wavelet low- and selected high-frequency sub-bands, random noise is removed using the multiscale NLM filter in both low- and high-frequency sub-bands without loss of image detail. The performance of the proposed method is compared to various existing methods on a set of push-broom-type sensor data acquired by Korean Multi-Purpose Satellite 3 (KOMPSAT-3) with severe stripe and random noise, and the results of the proposed method show significantly improved enhancement results over existing state-of-the-art methods in terms of both qualitative and quantitative assessments.

## 1. Introduction

The very high-resolution (VHR) satellite images acquired by a push-broom-type sensor are frequently contaminated by random and stripe noises. Push-broom-type sensors have been recently equipped in Korean Multi-Purpose Satellite 3 (KOMPSAT-3) (launched 2012), WoldView-2 (launched 2009), GeoEye-1 (launched 2008) and QuickBird-2 (launched 2001) and are subject to in-track striping without scan periodicity, since each line in the image is simultaneously acquired by a one-dimensional cross-track charge-coupled device (CCD) array. This type of sensor ensures a high signal-to-noise ratio (SNR) due to a longer dwell time than the whisk-broom-type sensor at the cost of random and stripe noises. Random noise is generated by the photoelectric effect of the imaging sensor, whereas stripe noise is generated by the differential variations in sensor sensitivity to perceived energy. Both types of noise are mixed together and significantly degrade the quality of push-broom-type satellite images [1,2].

Despite the pre-launch calibration of the satellite imaging sensor, in-flight calibration is additionally required for accurate image analysis. Therefore, removal of both random and stripe noise (or simultaneous denoising and destriping) is an important preprocessing step for both sensor calibration and image enhancement. In the past few decades, various denoising and/or destriping methods have been proposed to enhance satellite imagery.

Conventional denoising methods in the remote sensing field include total variation (TV)-based [3,4], wavelet-based [5,6] and non-local means (NLM) filtering-based [7,8]. Recently, improved versions of the denoising method provided better results than conventional denoising methods. The shape-adaptive DCT (SADCT) uses an intersection of confidence intervals to define the shape of the transformation support in a point-wise adaptive manner [9]. The block matching and three-dimensional (BM3D) filter places two-dimensional (2D) fragments of an input image into a three-dimensional (3D) array (groups), where transform coefficients are shrunk [10]. These methods are based on the well-known NLM filter [7]. Therefore, existing denoising methods [9,10] cannot preserve the details in the image. In the remote sensing field, another improved version was proposed to preserve the details using auxiliary images as the prior information of the noise-free frequency bands [11].

Along with the advancement of the denoising technique, many destriping methods were developed to remove the stripe noise in satellite images. Stripe noise introduces strong biases in the analysis of geometric structure, segmentation and registration. The conventional destriping methods can be categorized into three groups: (i) filtering-based; (ii) statistical matching-based; and (iii) variation model-based methods.

The filtering-based methods reduce the stripe noise in spatial and/or frequency domains using low-pass filters [12,13], shrinkage functions [14,15], the improved minimum noise fraction-based filter [16] and a spatial-spectral analysis-based filter [17], respectively. Although the filtering-based approach can provide a successful result using a simple computational structure, important structural information is removed together with the stripes, thereby leading to blurring and ringing artifacts.

The statistical matching-based approach uses two different matching criteria: moment and histogram. The moment-matching method corrects the stripe noise using the transformation of the sensor response function by assuming that input signals have the same mean and standard deviation [18,19]. Recently, improved versions of the moment-based method using either local or global image moment were proposed [20,21]. On the other hand, the histogram-matching method corrects the stripe noise using the modified nonlinear detector responses by assuming that input signals have the same probability density functions [22,23]. Although these methods can preserve the radiometric integrity of the satellite image data, they cannot avoid the limitation of completely removing the noise that cannot be statistically estimated.

The variation model-based methods are widely used for destriping, since they can preserve edges using the geometrical characteristics of the input signal. These directly use the geometrical features of the stripe noise to improve the performance of existing filtering-based and statistical matching-based methods. Shen *et al.* proposed a destriping method based on maximum *a posteriori* (MAP) with a Huber–Markov prior, which can be considered as an alternative between the isotropic total variation (TV) and Tikhonov regularization [24]. In particular, since stripes have clear directional signatures [25], an anisotropic TV is preferred to the isotropic ones [26]. Recently, Chang *et al.* proposed an advanced simultaneous destriping and denoising method using a unidirectional total variation and sparse representation model [27]. Yuan *et al.* proposed a spectral-spatial kernel regularized method [28]. These methods provide the best restoration results by selecting the optimum regularization parameter and *a prior* constraint. Although these methods were designed to avoid the restoration artifacts, the iterative regularization process takes a long processing time with high computational load [24,25,26,27,28].

The wavelet-based technique has received increasing attention in denoising and destriping in the remote sensing imagery [29]. Since the wavelet transform can decompose the input image into multiple, different scales, a simultaneous space-frequency analysis becomes possible. Donoho *et al.* proposed a wavelet-based denoising method using the hard- and/or soft-thresholding in the high-frequency wavelet sub-bands [30]. Torres *et al.* also used the wavelet transform to analyze and reduce the stripe noise by removing the wavelet coefficients of the high-frequency sub-band in the stripe direction [15]. However, Torres’s method cannot avoid undesired denoising artifacts in the process of the inverse wavelet transform with missing high-frequency components. Recently, advanced destriping methods tried to minimize the loss in high-frequency components by suppressing undesired artifacts [14,31,32]. Most wavelet-based methods assumed that the stripe noise is present in a directional high-frequency sub-band, while random noise is present in the entire frequency sub-bands in the wavelet domain. However, in the scanning process by the push-broom-type image sensor, the stripe noises are also observed in the low-frequency sub-band and cannot be completely removed by suppressing only high-frequency wavelet coefficients.

In order to preserve the radiometric integrity of satellite data, we present a novel image restoration framework that selectively combines destriping and denoising algorithms in the wavelet domain. More specifically, destriping and denoising methods in the proposed framework are improved versions of the wavelet-Fourier [32] and multiscale Super-resolution (SR) [33] methods, respectively. The wavelet-Fourier filtering optimally selects the frequency component in the wavelet sub-bands, and multiscale-based SR can restore the high-frequency signals by incorporating the patch similarity in the down-scaled space.

Based on the above-mentioned filtering methods, the proposed algorithm first separates the stripe and random noise using a wavelet-Fourier-based band-pass filter. Next, it estimates the noise visibility function (NVF) data [34] in the filtered wavelet low-frequency sub-band that is free from stripe effects. The NVF data represents the amount of local variance and is used to separate stripe noise from the high-frequency details that should be preserved in the wavelet high-frequency sub-bands. In order to remove the random noise without blurring artifacts, the original version of the NLM filter [7] is modified under the multiscale framework. Since the proposed multiscale NLM filter utilizes more patch redundancy in the down-scaled space, it guarantees that the most similar patch can be always selected. Therefore, the proposed denoising method can remove the random noise with minimum blurring artifacts using the weighted summation of the selected similar patches. For this reason, the proposed method can provide promising denoising and destriping performance by selectively performing the filtering-based noise removal in the specific sub-bands of the wavelet domain containing both stripe and random noise.

The major contributions of this work are two-fold: (i) it can remove the mixed stripe and random noise using a combined destriping and denoising filter in the wavelet domain; and (ii) it can minimize the blurring artifacts using the proposed multiscale version of the NLM filter. The proposed method is compared to various existing methods in the sense of both objective and subjective assessments using a set of satellite images acquired by Korean Multi-Purpose Satellite 3 (KOMPSAT-3) equipped with a push-broom-type sensor. Through comparative experiments, the proposed method demonstrates that it can efficiently remove mixed noise. In additional experiments, the proposed method is applied to the pan-sharpening process, and it can provide high-quality satellite color images from noisy multispectral and panchromatic inputs.

The rest of this paper is organized as follows. Section 2 describes the image degradation model of the push-broom-type imaging sensor. Section 3 presents the combined wavelet-Fourier and multiscale NLM filters in the wavelet domain. Section 4 summarizes experimental results, and Section 5 concludes the paper.

## 2. Image Degradation Model

In the process of acquiring a two-dimensional (2D) image using the push-broom-type sensor, the detection error of CCD generates the stripe and random noise. Specifically, stripe noise is generated by different inter-line sensitivities, whereas random noise is generated by the photoelectric effect of the imaging sensor. In order to propose a simultaneous destriping and denoising method, a combined stripe and random noise degradation model is presented in this section.

Let *x* be the CCD detector index (sample index) in the cross-track direction and *y* the dimension index of obtained one-dimensional (1D) signal (line index) in the along-track direction. The image degradation model of the push-broom-type sensor is defined as:

(1) $$g\left(x,y\right)=f\left(x,y\right)+\eta \left(x,y\right),\;\;\;\text{for}\;x=1,\ldots ,N_{x},\;\;y=1,\ldots ,N_{y}$$

where *g*(*x*, *y*) is a noisy, observed image, *f*(*x*, *y*) the noise-free, original image and η(*x*, *y*) the noise. *N_x_* is the number of sample, that is the number of 1D CCD detectors in the cross-track direction, and *N_y_* the number of lines acquired in the along-track direction.

Stripe noise in the push-broom-type sensor is generally aperiodic and mixed with random noise. Therefore, the noise term of Equation (1) can be rewritten as [2]:

(2) g(x,y)=f(x,y)+η(x,y,f(x,y))

where:

(3) $$\eta \left(x,y,f\left(x,y\right)\right)=\eta_{S}\left(x,f\left(x,y\right)\right)+\eta_{R}\left(x,y\right)$$

According to Equation (3), the combined noise term η(*x*, *y*, *f*(*x*, *y*)) consists of stripe noise η_**S**_(*x*, *y*, *f*(*x*, *y*)) and random noise η_**R**_(*x*, *y*). The stripe noise is generated by the differential variations in sensor sensitivity and depends on the original image *f*(*x*, *y*), as well as the sample indices. For this reason, we assume that the stripe noise generated in the push-broom-type sensor has a unidirectional pattern. On the other hand, the random noise is generated by the photoelectric effect of the sensor and can be modeled as an adaptive white Gaussian (AWGN) random process with zero-mean and variance σ^2^.

## 3. Correction of Satellite Sensor Data

In this section, we describe a simultaneous stripe and random noise removal method using a combined wavelet-Fourier filtering and multiscale non-local means (NLM) filter in the wavelet domain. In the wavelet domain, the image restoration problem in Equation (2) can be expressed as [35]:

(4) $$\tilde{g}\left(x,y\right)=w\left(x,y\right)+{\tilde{\eta }}_{S}\left(x,w\left(x,y\right)\right)+{\tilde{\eta }}_{R}\left(x,y\right)$$

where $\tilde{g}\left(x,y\right)$ represents the wavelet transform of noisy, observed image, *w*(*x*, *y*) the wavelet transform of the noise-free, original image, ${\tilde{\eta }}_{S}\left(x,w\left(x,y\right)\right)$ the wavelet version of stripe noise and ${\tilde{\eta }}_{R}\left(x,y\right)$ the wavelet version of random noise.

Given Equation (4), the goal of the proposed work is to estimate the original wavelet coefficient *w*(*x*, *y*) by destriping and denoising without the loss of image details. However, estimation of the original signal from the corrupted version by both stripe and random noise is an ill-posed problem. Practically, removal of random noise results in a damaged pattern of the stripe noise, which makes the following destriping process more difficult [27]. On the other hand, removal of striping noise may change the statistical distribution of random noise, and the removal of a non-Gaussian random noise becomes more difficult. Assuming that the stripe noise in the push-broom-type image sensor is unidirectional in the along-track direction, stripe noise exists only in the low-frequency wavelet sub-bands, including the LL and HL sub-bands. For this reason, the selective filtering in the LL and HL sub-bands minimizes the statistical distribution of random noise coefficients contained in the wavelet LH and HH sub-bands.

Based on the above-mentioned noise property, the proposed method performs destriping and denoising using combined wavelet-Fourier filtering and multiscale NLM filtering in the wavelet domain. The restored image in the wavelet domain can be obtained using the least-squares optimization as:

(5) $$\hat{w}\left(x,y\right)=\arg\min_{w(x,y)}\sum_{\left(m,n\right)\in \Omega_{D}}{\left[\text{FT}\left\{{\tilde{g}}_{D}^{P}\left(m,n\right)-w\left(x,y\right)\right\}\right]}^{2}S^{P}\left(m,n,x,y\right)$$

where Ω_**D**_ represents a local region in the **D** times down-sampled scale, ${\tilde{g}}_{D}^{P}\left(m,n\right)$ the local patches of $\tilde{g}\left(x,y\right)$ corresponding to Ω_**D**_, FT {·} the Fourier filtering operator that is the proposed combined wavelet-Fourier filter to remove the stripe noise in the LL and HL sub-bands, *w*(*x*, *y*) is the original wavelet coefficients containing all sub-band, **S^P^**(*m*, *n*, *x*, *y*) the similarity weighting value between the local patches ${\tilde{g}}_{D}^{P}\left(m,n\right)$ in the down-scaled space and the corresponding patch in $\tilde{g}\left(x,y\right)$ and the superscript **P** denotes a patch.

The solution of the least-squares estimation in Equation (5) is given as:

(6) $$\hat{w}\left(x,y\right)=\frac{\sum_{\left(m,n\right)\in \Omega_{D}}S^{P}\left(m,n,x,y\right)\text{FT}\left\{{\tilde{g}}_{D}^{P}\left(m,n\right)\right\}}{\sum_{\left(m,n\right)\in \Omega_{D}}S^{P}\left(m,n,x,y\right)}$$

**Figure 1:**
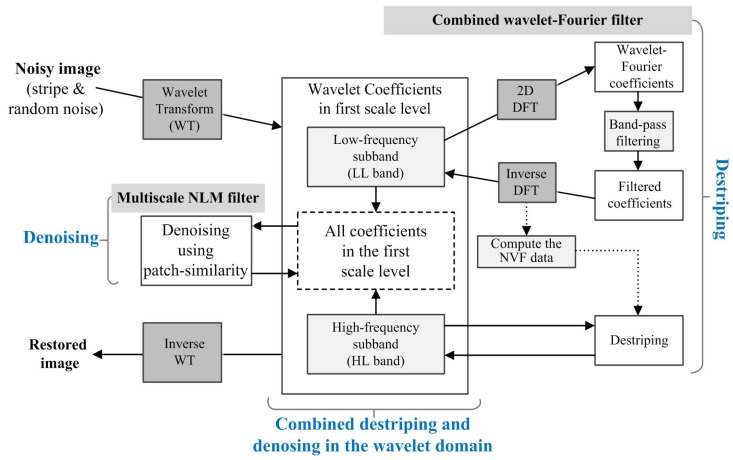
Block-diagram of the proposed combined wavelet-Fourier and multiscale non-local means (NLM) filtering method.

Therefore, the solution of the least-squares estimation can remove both stripe and random noise using the combined wavelet-Fourier filter and multiscale NLM filter in the wavelet domain. More specifically, the unidirectional stripe noise in the HL sub-band can be removed using the estimated NVF data [34] and the wavelet-Fourier-based band-pass filtered wavelet LL sub-band. Next, the proposed method removes random noise in all four wavelet sub-bands using multiscale NLM filtering. As a result, it can preserve image details by incorporating the patch similarity in the down-scaled space. The block-diagram of the proposed method is shown in Figure 1.

### 3.1. Combined Wavelet-Fourier Filtering

The wavelet transform is used to analyze a signal using a set of band-pass filters that decomposed the noisy image *g*(*x*, *y*) into low-frequency wavelet coefficients, ${\tilde{g}}_{LL}\left(x,y\right)$ called the LL band, and three directional high-frequency coefficients, ${\tilde{g}}_{LH}\left(x,y\right)$, ${\tilde{g}}_{HL}\left(x,y\right)$ and ${\tilde{g}}_{HH}\left(x,y\right)$, respectively called the LH, HL and HH bands. The mathematical description of the wavelet transform is given as [35]:

(7) $${\tilde{g}}_{LL}\left(p_{0},x,y\right)=\frac{1}{\sqrt{MN}}\sum_{x=0}^{M-1}\sum_{y=0}^{N-1}g\left(x,y\right)\varphi_{p_{0},x,y}\left(x,y\right)$$

and:

(8) $${\tilde{g}}_{q}\left(p,x,y\right)=\frac{1}{\sqrt{MN}}\sum_{x=0}^{M-1}\sum_{y=0}^{N-1}g\left(x,y\right)\psi_{p,x,y}^{q}\left(x,y\right),\;\text{for}\;q\in \left\{LH,HL,HH\right\}$$

where the subscript *p*_0_ represents an arbitrary starting scale, ${\tilde{g}}_{LL}\left(p_{0},x,y\right)$ the approximation coefficients of *g*(*x*, *y*) at scale *p*_0_, ${\tilde{g}}_{q}\left(p,x,y\right)$ one of three directional wavelet coefficients and φ(*x*, *y*) and ψ(*x*, *y*) respectively the 2D scaling and wavelet functions [30].

**Figure 2:**
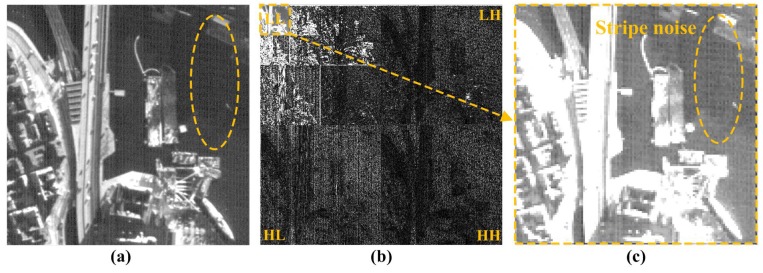
(**a**) A Korean Multi-Purpose Satellite 3 (KOMPSAT-3) image acquired by the push-broom-type sensor with mixed stripe and random noise; (**b**) three-level wavelet decomposition results using symmetrical wavelet (symlet) filters; and (**c**) the eight-times enlarged version of the LL sub-band.

Recently, a wavelet-based destriping method to enhance a push-broom-type sensor image utilizes the non-uniformly-spaced parallel pattern of stripe noise in the HL wavelet sub-band with the along-track direction [14,31,32]. The push-broom-type sensor generates the stripe noise if adjacent CCD detectors have non-uniform responses. In this case, the stripe noises are observed in the LL wavelet sub-band, as well as high-frequency sub-bands, as shown Figure 2b,c.

**Figure 3:**
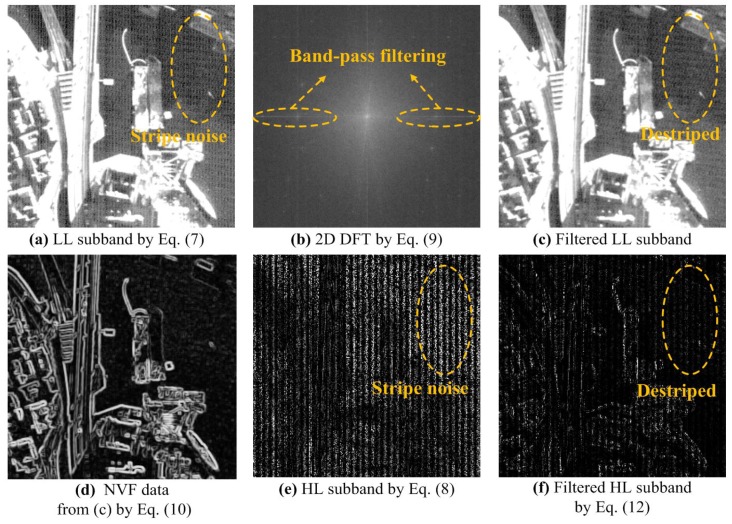
Step-by-step results of the combined wavelet-Fourier filtering for the KOMPSAT-3 image shown in Figure 2: (**a**) the LL sub-band of one-level wavelet decomposition; (**b**) 2D DFT of (**a**); (**c**) the destriped LL sub-band using a Fourier band-pass filter; (**d**) NVF data generated from the filtered LL sub-band; (**e**) the HL sub-band with mixed stripe and random noise; and (**f**) the destriped HL sub-band using the NVF data.

The proposed method first generates auxiliary data from the combined wavelet-Fourier filtered version of the LL sub-band. A rigorous description of the NVF data will be given at the end of this subsection. Next, the stripe noise coefficients in the HL sub-band are removed using the NVF data generated in the previous steps. Specifically, the proposed combined wavelet-Fourier filtering method consists of three steps: (i) band-pass filtering at LL sub-band, as shown in Figure 3b; (ii) NVF data generation from the filtered LL sub-band, as shown in Figure 3d; and (iii) suppression of stripe noise coefficients in the HL sub-band using the NVF data, as shown in Figure 3f. Step-by-step results of the combined wavelet-Fourier filtering are summarized in Figure 3.

Figure 3a shows the LL sub-band of one-level wavelet transform, and Figure 3b shows the results of combined wavelet-Fourier transform as:

(9) $${\tilde{G}}_{LL}\left(u,v\right)=\sum_{x=0}^{M-1}\sum_{y=0}^{N-1}{\tilde{g}}_{LL}\left(x,y\right)\text{exp}\left\{-j2\pi \left(\frac{xu}{M}+\frac{yv}{N}\right)\right\}$$

where (*u*, *v*) represents the 2D spatial frequency coordinate and ${\tilde{g}}_{LL}$ represents the wavelet transformed LL sub-band.

The wavelet-Fourier power spectrum ${\tilde{G}}_{LL}$ includes the frequency components corresponding to the stripe noise and can be removed by the band-pass filter [35] as in the first step of combined wavelet-Fourier filtering. Since the stripe noise of a push-broom-type sensor in the LL and LH wavelet sub-bands has a unidirectional pattern, we perform the wavelet-Fourier-based band-pass filtering in the LL sub-band to preserve the high-frequency signals contained in the HL, LH and HH sub-bands. Figure 3c shows the inverse DFT of the filtered wavelet-Fourier power spectrum. In the second step of the proposed method, in order to remove the stripe noise in the HL sub-band, the NVF data [34] are estimated from the filtered LL sub-band as:

(10) $$\Lambda_{LL}\left(x,y\right)=\frac{1}{1+\phi var(x,y)}$$

where the tuning parameter ϕ is chosen, so that Λ_*LL*_ distributes as uniformly as possible in [0,1], and var(*x*, *y*) is the variance of a 5 × 5 local region centered at (*x*, *y*) in the filtered LL sub-band ${\hat{g}}_{LL}$ defined as:

(11) $$\text{var}\left(x,y\right)=\frac{1}{25}\sum_{(x,y)\in \Omega }{\left\{{\hat{g}}_{LL}\left(x,y\right)-{\text{mean}}_{xy}\right\}}^{2}$$

where Ω represents a 5 × 5 support centered at (*x*, *y*), ${\hat{g}}_{LL}\left(x,y\right)$ the filtered wavelet LL coefficients and mean_*xy*_ the local mean of the support Ω. Since the NVF data Λ_*LL*_ estimated by Equation (11) contain the local variance of the LL sub-band, these can be used to remove the stripe noise in the wavelet-Fourier filtered HL sub-band as:

(12) $${\hat{g}}_{HL}\left(x,y\right)=\left\{1-\Lambda_{LL}\left(x,y\right)\right\}{\tilde{g}}_{HL}\left(x,y\right)$$

where ${\hat{g}}_{HL}$ represents the filtered HL sub-band without stripe noise, Λ_*LL*_ the NVF data generated by Equation (10) and ${\tilde{g}}_{HL}$ the noisy HL sub-band with mixed stripe and random noise coefficients.

**Figure 4:**
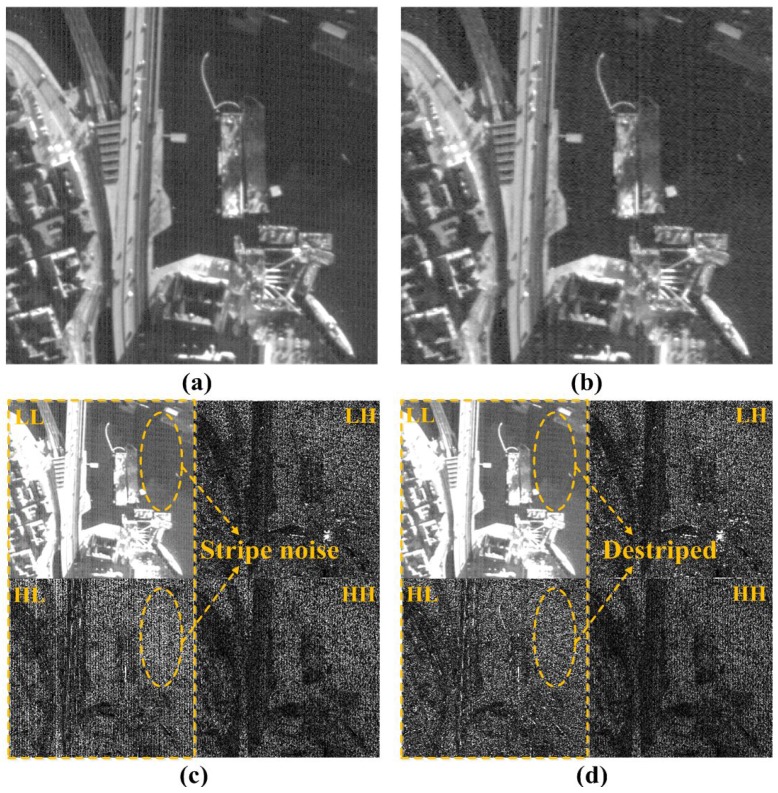
Result of combined wavelet-Fourier filtering: (**a**) an input KOMPSAT-3 image acquired by the push-broom-type sensor containing mixed stripe and random noise; (**b**) the result of destriping using the combined wavelet-wavelet-Fourier filtering; (**c**) one-level wavelet transform of (**a**); and (**d**) one-level wavelet transform of (**b**). In both LL and HL sub-bands, stripe noise is significantly reduced.

Results of the proposed combined wavelet-Fourier filtering are shown in Figure 4. Since the NVF data are generated using the local variance from filtered LL sub-band as given in Equation (10), they have the detail of the signal without stripe noise. Especially, these data can accurately separate stripe noise and signal details using the space-frequency localization property of the wavelet transform. For this reason, the proposed method removes the stripe noise components in the HL sub-band without losing the details of the signal.

**Figure 5:**
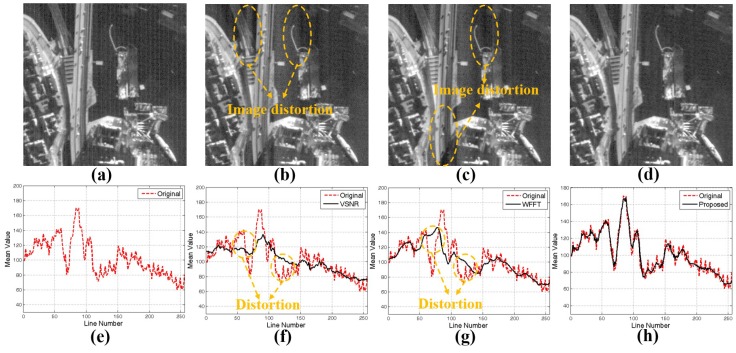
Results of three destriping methods using a KOMPSAT-3 image: (**a**) the input image; (**b**) result of the variational stationary noise remover (VSNR) method [25]; (**c**) result of the wavelet-Fourier filtering (WFFT) method [32]; (**d**) result of the proposed method; (**e**–**h**) mean cross-track profiles of the input image, the VSNR result the WFFT result and the proposed result.

Figure 5 shows comparative stripe noise removal performance using variational stationary noise remover (VSNR) [25] and wavelet-Fourier filtering (WFFT) [32] using the mixed noisy image.

Figure 5a shows a mixed noisy version of the original KOMPSAT-3 image containing stripe and random noise. In Figure 5b,c, although VSNR and WFFT destriping methods can remove stripe noise in the high- and low-frequency regions, undesired restoration artifacts remain in the low-frequency regions. On the other hand, the proposed destriping method can successfully remove the stripe noise without undesired image distortion, as shown in Figure 5d. Figure 5f–h shows the mean cross-track profiles of VSNR, WFFT and the proposed destriping methods. As shown in the figures, the proposed method provides the best fitted curves to the original image. Figure 5 shows that the proposed method can remove the stripe noise without undesired restoration artifacts. The pseudo code of the proposed wavelet-Fourier filtering algorithm is shown in Algorithm 1.

### 3.2. Multiscale Non-Local Means Filtering

In the wavelet domain, filtered LL and HL sub-bands and LH and HH sub-bands contain only random noise, η_**R**_(*x*, *y*). The random noise of the push-broom-type sensor is assumed to have a Gaussian distribution with zero-mean and variance σ^2^. A simple thresholding method can reduce the random noise using the hard threshold value of 2σ^2^. However, it generates a Gibbs-like phenomenon by cancellation of the wavelet coefficients lower than the original wavelet coefficients value.
**Algorithm 1** Combined wavelet-Fourier filtering **input:** noisy image *g*
    support windows size Ω = 5     tuning parameter ϕ =500 **output:** destriped wavelet sub-bands (${\hat{g}}_{LL}$, ${\hat{g}}_{HL}$) 1: **Step 1:**one-level wavelet transform using symmetry wavelet (sym4) 2:[wa,wh,wv,wd] = dwt2(g ,′sym4′)Equations (7) and (8) 3: **Step 2:**wavelet-Fourier filtering 4:waFFT = fftshift(fft2(wa))Equation (9) 5:waFFT = band pass filtering(waFFT) using [35] 6:waFFT → **Output:**
${\hat{g}}_{LL}$ 7: **Step 3:**compute the NVF data from step 2 8:compute the NVF data from step 2 mean = filter2(ones(Ω),waFFT)/ prod(Ω)^2^Equation (11) 9:var = filter2(ones(Ω),waFFT^2^/ prod(Ω)^2^-mean^2^Equation (11) 10:nvfdata = 1./(1+ϕ*var)Equation (10) 11: **Step 4:**HL sub-band filtering 12:wv = wv.*(1-nvfdata)Equation (12) 13:wv → **Output:**
${\hat{g}}_{HL}$


**Figure 6:**
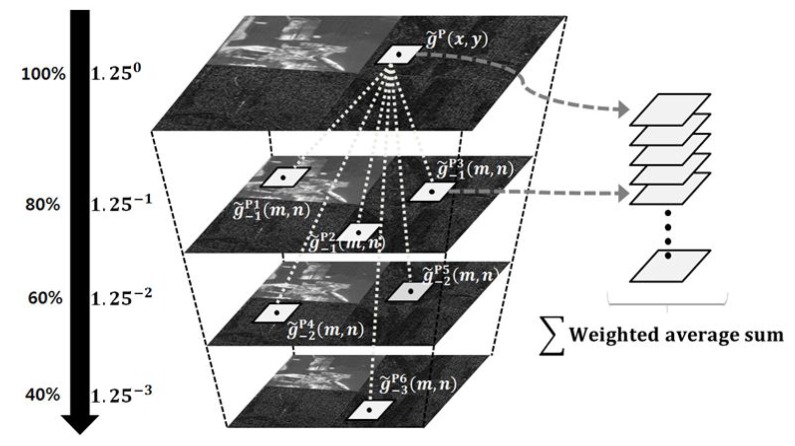
Illustration of a patch-searching process in the proposed multiscale NLM filtering.

To solve these problems, the original version of the NLM filter is modified under a multiscale framework to remove Gaussian random noise with minimum undesired artifacts. The proposed multiscale NLM filter consists of three steps: (i) generation of down-scaled wavelet sub-bands using the bicubic interpolation kernel; (ii) computation of the weighting value based on the similarity between the input and corresponding patches in the down-scaled wavelet sub-bands; and (iii) weighted summation of patches to remove the Gaussian random noise. The overall process of the multiscale NLM filtering is illustrated in Figure 6.

In the first step, the wavelet transform coefficients without stripe noise is down-scaled by 1.25^**D**^ times to generate ${\tilde{g}}_{D}\in \left\{{\hat{g}}_{LL},{\tilde{g}}_{LH},{\tilde{g}}_{HL},{\hat{g}}_{HH}\right\}$, for $D=\left\{-1,-2,-3\right\}$, using the bicubic interpolation kernel [35]. Since the bicubic interpolation kernel performs low-pass filtering, it decreases the noise variance and guarantees searching sufficiently similar patches [33]. Next, the similarity weighting value is computed in the down-scaled space as:

(13) $$S^{P}\left(m,m,x,y\right)=\exp\left(-\frac{{∥{\tilde{g}}_{D}^{P}\left(m,n\right)-{\tilde{g}}^{P}\left(x,y\right)∥}_{G}^{2}}{h}\right)$$

where ${\tilde{g}}_{D}^{P}\left(m,n\right)$ represents the coefficients of the patch centered at the location of ${\tilde{g}}_{D}\left(x,y\right)$ and ${\tilde{g}}^{P}\left(x,y\right)$ the coefficients of the patch centered at the location of $\tilde{g}\left(x,y\right)$ of the original scale. The parameter **G** is a Gaussian kernel that controls the exponential decay in the weighting computation, and *h* is a global filter parameter [7].

As given in Equation (13), the proposed method uses the exponential function instead of a hard threshold for determining adaptively optimal weighting values. If a pair of patches is not similar, the Euclidean distance decreases, and the similarity value becomes smaller. Based on the property of the similarity weighting value, it is guaranteed that the most similar patch can be always selected.

Another issue of the proposed filtering method is how to determine the global filter parameter *h*. Since the low-pass filtering property of the bicubic interpolation kernel decreases the noise variance in the down-scaled images, the similarity weighting value should be correspondingly decreased as the down-scaling ratio becomes larger. Likewise, we estimate the new adaptive global filter parameter for each down-scaled image.

To estimate the noise variance σ^2^ from the noisy wavelet coefficients, a robust median estimator is used from the finest scale wavelet coefficients (*i.e.*, the HH sub-band) as [30]:

(14) $$\sigma^{2}=\frac{\text{median}\left(\left|{\hat{g}}_{HH}\right|\right)}{0.6745}$$

The similarity weighting value is estimated as:

(15) $$S^{P}\left(m,m,x,y\right)=\text{exp}\left(-\frac{{∥{\tilde{g}}_{D}^{P}\left(m,n\right)-{\tilde{g}}^{P}\left(x,y\right)∥}_{G}^{2}}{1.25^{D}\sigma^{2}}\right)$$

In the last step, the restored wavelet coefficients $\hat{w}\left(x,y\right)$ is obtained by the weighted averaging sum as:

(16) $$\hat{w}\left(x,y\right)=\frac{\sum_{\left(m,n\right)\in \Omega_{D}}S^{P}\left(m,m,x,y\right){\tilde{g}}_{D}^{P}\left(m,n\right)}{\sum_{\left(m,n\right)\in \Omega_{D}}S^{P}\left(m,m,x,y\right)}$$

where $S^{P}\left(m,m,x,y\right)$ stands for the patch similarity weight value computed using Equation (16) and ${\tilde{g}}_{D}^{P}\left(m,n\right)$ represents the patch corresponding to $\tilde{g}\left(x,y\right)$, including random noise in the down-scaled space.

**Figure 7:**
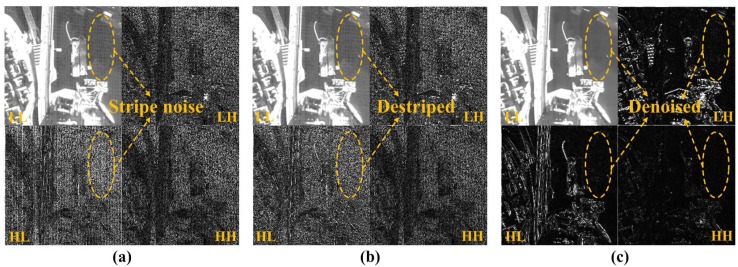
Results of the proposed multiscale NLM filtering in the wavelet domain: (**a**) one-level wavelet transform with stripe and random noise; (**b**) destriped LL and HL wavelet sub-bands; and (**c**) denoised results of all wavelet sub-bands.

Results of denoising in the wavelet domain are shown in Figure 7. As shown in Figure 7, the proposed method can selectively remove the mixed stripe and random noise in the wavelet domain.

**Figure 8:**
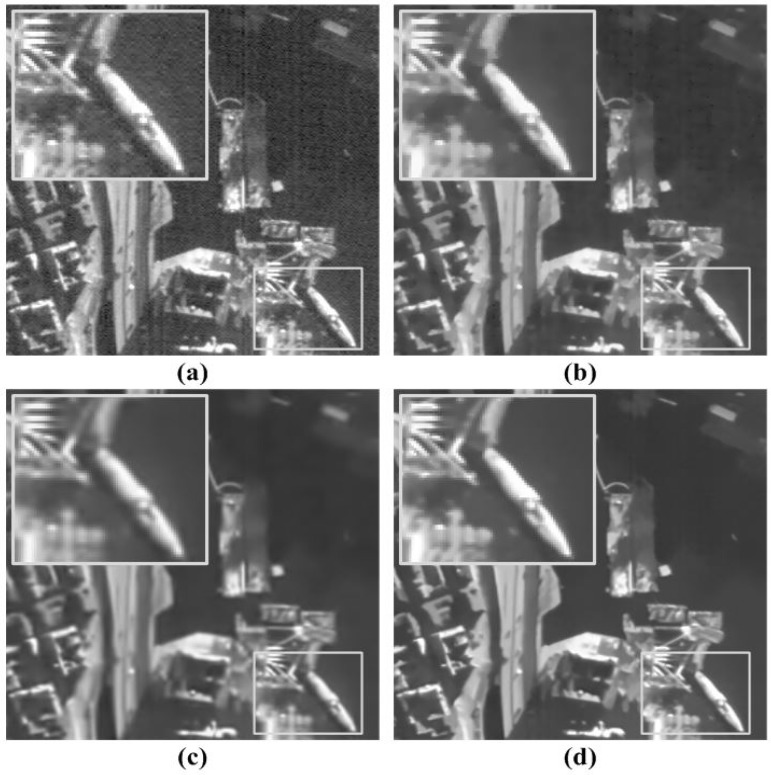
Results of three denoising methods using a KOMPSAT-3 image: (**a**) the input image; (**b**) result of the total variation (TV) method [4]; (**c**) result of the spares representation over learned dictionary (SLD) method [36]; and (**d**) result of the proposed method.

The proposed denoising algorithm utilizes more patch redundancy in the multiscale space [33] than the in-scale, weighted averaging method [7]. For this reason, it can preserve the high-frequency details without the undesired blurring artifact in removing random noise. The objective performance is compared to existing denoising methods, as shown in Figure 8.

Figure 8a shows a destriped version of the original KOMPSAT-3 image containing only random noise. To evaluate the objective performance, the proposed denoising method is compared to the TV [4] and spares representation over learned dictionary (SLD) [36] methods. The regularization parameter in the TV method is set to be 30, and the SLD method uses default parameters recommended in [36]. The noise variance is equal to 15. Figure 8b provides the smoothed denoising result at the cost of blurring due to the regularization process. Figure 8c shows the denoising result with a different type of blurring artifact. On the other hand, the proposed method can successfully remove the random noise without the undesired blurring artifact, as shown in Figure 8d.

The pseudo code of the proposed multiscale NLM filtering algorithm is shown in Algorithm 2. The finally destriped and denoised image is obtained using the inverse wavelet transform of the restored wavelet coefficients [35].
**Algorithm 2** Multiscale non-local means filtering **input:** wavelet sub-bands $\tilde{g}\in \left\{{\hat{g}}_{LL},{\tilde{g}}_{LH},{\hat{g}}_{HL},{\tilde{g}}_{HH}\right\}$
    Gaussian kernel **G** in [7], ratio of similarity windows size f=2     down-scaled ratio **D** = {−1, −2, −3} **output:** denoised wavelet sub-bands $\hat{g}$ 1: **Step 1:**generate the down-scaled wavelet sub-bands using bicubic kernel [35] 2:**for D** ← 1 to 3 3: subImg(1,**D**) = struct(layer,[]) 4: subImg(1,**D**).layer = imresize(
$\tilde{g}$, $1.25^{\left(-D\right)}$, bicubic)In Figure 5 5:**end** 6: **Step 2:**compute the similarity weighting value in down-scaled wavelet sub-bands 7:input patch ${\tilde{g}}^{P}\left(x,y\right)$ → W1, current similarity patch ${\tilde{g}}_{D}^{P}\left(m,n\right)$ → W2 8:distance between W1 and W2 ${∥{\tilde{g}}_{D}^{P}\left(m,n\right)-{\tilde{g}}^{P}\left(x,y\right)∥}_{G}^{2}$ → dEquation (11) 9:weight value $S^{P}$ → s, initial average → 0 10:**for**
m,n ← searching windows size in [7, **D** ← 1 to 3 11:W2 = subImg(1,**D**).layer(-f+m:f+m,-f+n:f+n) 12:d = sum(sum(**G**.*(W1-W2).*(W1-W2))) 13:$\sigma^{2}\leftarrow \mathrm{median}\left(\left|{\hat{g}}_{HH}\right|\right)/0.6745$Equation (14) 14:s = exp(-d/($\sigma^{2}$*($1.25^{\left(-D+1\right)}$
)))Equation (15) 15: **Step 3:**weighted average sum to remove the random noiseEquation (15) 16:average = average+(s*subImg(1,**D**).layer(m,n))Equation (16) 17:average → **Output:**
$\hat{g}$ 18:**end**


## 4. Experiments and Discussion

**Figure 9:**
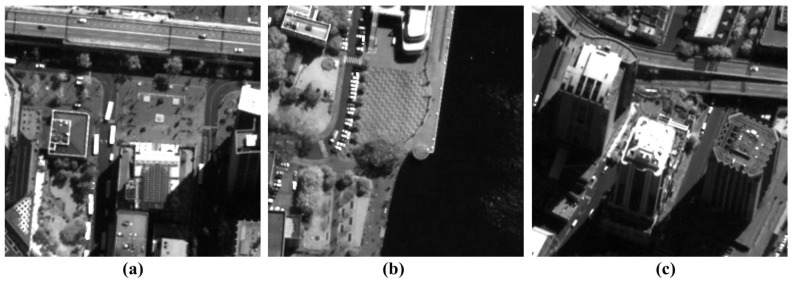
QuickBird-2 test images: (**a**–**c**) are acquired by QuickBird-2 satellite system in the panchromatic band without stripes and random noise.

**Figure 10:**
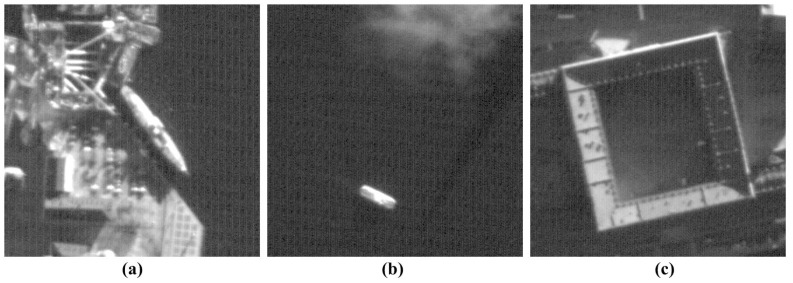
KOMPSAT-3 test images: (**a**–**c**) are acquired by KOMPSAT-3 satellite system in the panchromatic band with stripes and random noise.

In this section, we test the proposed method on real VHR satellite images acquired by two different push-broom-type sensors to evaluate the denoising performance. One set of test images is acquired by QuickBird-2, as shown in Figure 9, and the other by KOMPSAT-3, as shown in Figure 10.

For the comparison, wavelet-Fourier filtering (WFFT) [32], the variational stationary noise remover (VSNR) [25] and the wavelet-Fourier adaptive filter (WFAF) [31] are used to remove stripe noise, and wavelet hard-threshold (HardTh) [30], total variation (TV) [4], bivariate shrinkage functions (BiShrink) [5] and spares representation over learned dictionary (SLD) [36] methods are used to remove random noise. In the experiment, each denoising algorithm uses the parameters that give the visually best result. In the proposed method, the parameters of combined wavelet-Fourier filtering given in Algorithm 1, the size of the supporting window Ω and the tuning parameter ϕ are set to five and 500, respectively. In Algorithm 2, the similarity window size *f* of multiscale NLM filter is set to two, and the down-scaling level **D** of wavelet sub-bands $\tilde{g}\in \left\{{\hat{g}}_{LL},{\tilde{g}}_{LH},{\hat{g}}_{HL},{\tilde{g}}_{HH}\right\}$ is set to three. The wavelet transform scale level is one, and the noise variance is estimated by the robust median estimator [30].

To compare the qualitative performance, we used several qualitative and quantitative assessment measures, including peak signal-to-noise ratio (PSNR), noise reduction ratio (NR) [13], inverse coefficient of variation (ICV) [22], universal image quality index (UIQI) [37], Q-metric [38] and structural similarity (SSIM) [39]. The higher quality-score of PSNR, SSIM, UIQI, Q-metric, NR and ICV means higher image quality. NR , ICV and Q-metric are no-reference quality assessment measures, whereas PSNR, UIQI and SSIM require the reference image.

### 4.1. Simulated Experiment Results

**Figure 11:**
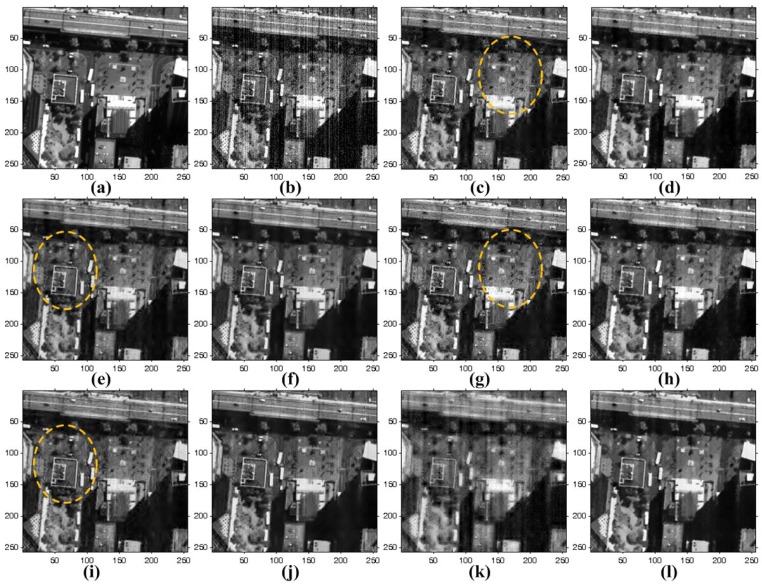
Results of various enhancement methods using the simulated QuickBird-2 image of Figure 9a: (**a**) noise-free image; (**b**) simulated degraded image; (**c**) WFFT-hard-threshold (HardTh); (**d**) WFFT-TV; (**e**) WFFT-bivariate shrinkage functions (BiShrink); (**f**) WFFT-SLD; (**g**) VSNR-HardTh; (**h**) VSNR-TV; (**i**) VSNR-BiShrink; (**j**) VSNR-SLD; (**k**) wavelet-Fourier adaptive filter (WFAF); and (**l**) the proposed method.

**Figure 12:**
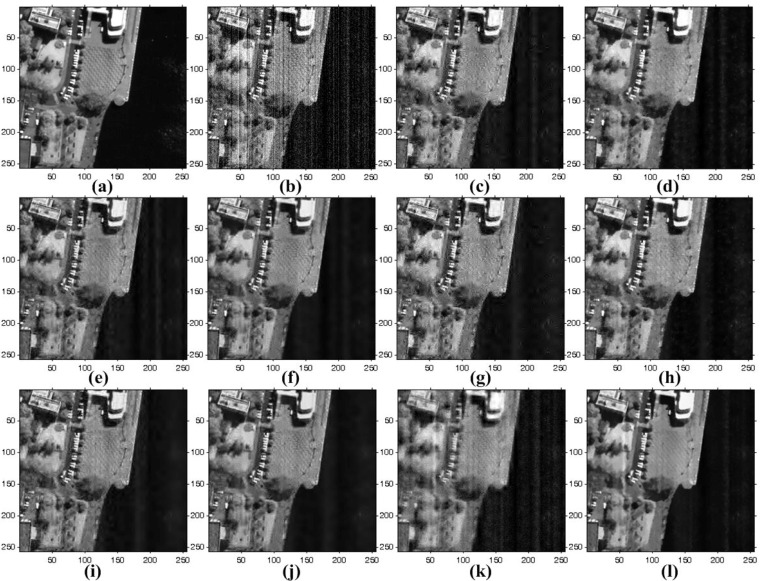
Results of various enhancement methods using the simulated QuickBird-2 image of Figure 9b: (**a**) noise-free image; (**b**) simulated degraded image; (**c**) WFFT-HardTh; (**d**) WFFT-TV; (**e**) WFFT-BiShrink; (**f**) WFFT-SLD; (**g**) VSNR-HardTh; (**h**) VSNR-TV; (**i**) VSNR-BiShrink; (**j**) VSNR-SLD; (**k**) WFAF; and (**l**) the proposed method.

In order to add stripe and random noise by simulation, we used three noise-free QuickBird-2 images. The performance of the proposed method is compared to existing methods, as shown in Figure 11 and Figure 12. For the experiment, WFFT and VSNR are preformed to remove the stripe noise, and random noise is then removed using HardTh, TV, BiShrink and SLD. WFAF is additionally used to remove both stripe and random noise simultaneously.

Figure 11a shows a noise-free image. After adding the stripe noise at every line, the zero-mean white Gaussian noise with standard deviation σ = 25 is used to obtain the simulated version of the noisy image, as shown in Figure 11b. Although the WFFT, VSNR and WFAF methods can remove stripe noise in the high-frequency region, stripe artifacts remain in the low-frequency region. In the highlighted regions with dotted circles, as shown in Figure 11c,e,g,i, wavelet-based denoising methods show a Gibbs-like phenomenon generated by clipping out the wavelet coefficients. In Figure 11d,h, the WFFT-TV and VSNR-TV methods still show noise artifacts in the denoising process, since the TV method lays too much emphasis on the smoothness of noisy images. Figure 11f,j shows the results where stripe and random noise were successfully removed by the WFFT-SLD and VSNR-SLD methods. However, the SLD method results in patch mismatching error in the trained data. The WFAF method cannot completely remove stripes along the horizontal direction, as shown in Figure 11k. As shown in Figure 11 and Figure 12, although the mixed noise removal performance depends on the strength of both stripe and random noise, the proposed method can provide improved enhancement results over existing denoising and destriping methods.

**Figure 13:**
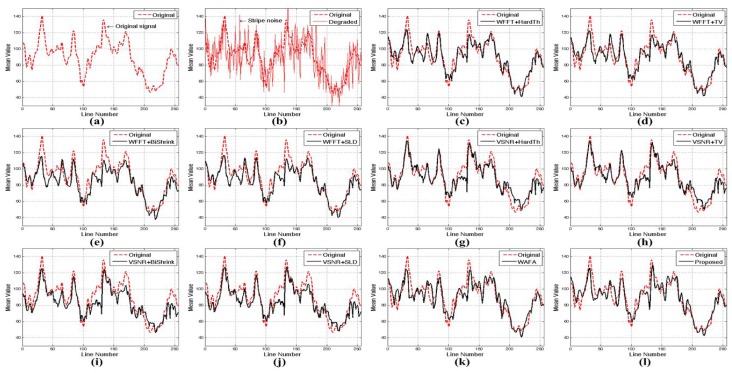
Mean cross-track profiles of Figure 11: (**a**) noise-free image; (**b**) simulated degraded image; (**c**) WFFT-HardTh; (**d**) WFFT-TV; (**e**) WFFT-BiShrink; (**f**) WFFT-SLD; (**g**) VSNR-HardTh; (**h**) VSNR-TV; (**i**) VSNR-BiShrink; (**j**) VSNR-SLD; (**k**) WFAF; and (**l**) the proposed method.

**Figure 14:**
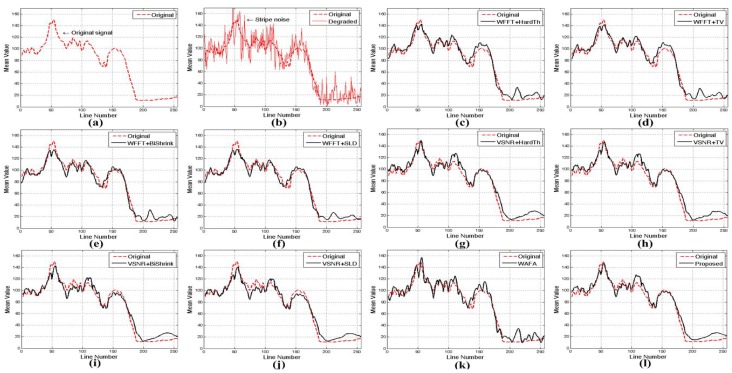
Mean cross-track profiles of Figure 12: (**a**) noise-free image; (**b**) simulated degraded image; (**c**) WFFT-HardTh; (**d**) WFFT-TV; (**e**) WFFT-BiShrink; (**f**) WFFT-SLD; (**g**) VSNR-HardTh; (**h**) VSNR-TV; (**i**) VSNR-BiShrink; (**j**) VSNR-SLD; (**k**) WFAF; and (**l**) the proposed method.

Figure 13 and Figure 14 show the mean cross-track profiles of different denoising methods. As shown in the figures, the proposed method provides the most similar curves to the original noise-free image.

The PSNR, SSIM and UIQI values of the simulated noisy images shown in Figure 9 are computed for ten different methods as summarized in Table 1, Table 2 and Table 3. Based on the tables, the proposed method also shows the best performance in the sense of the objective measure. We compared the processing time to process an input noisy image of a size of 256 × 256, as shown in Figure 8, using a personal computer with a 3.60-GHz CPU and 64 GByte of RAM. The VSNR-SLD and VSNR-TV methods take 11.65 and 2.94 s, respectively. The proposed method takes only 2.91 s and produces significantly improved denoising results than existing state-of-the-art methods.

### 4.2. Real Experiment Results

**Figure 15:**
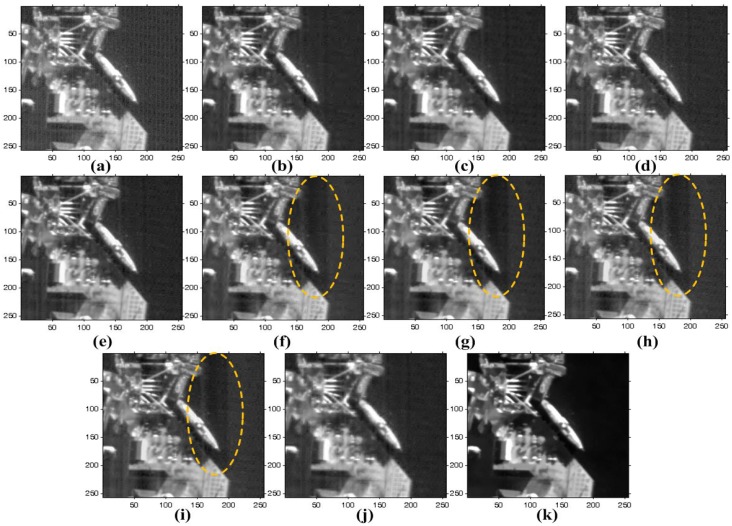
Results of various enhancement methods using the KOMPSAT-3 image of Figure 10a: (**a**) original image; (**b**) WFFT-HardTh; (**c**) WFFT-TV; (**d**) WFFT-BiShrink; (**e**) WFFT-SLD; (**f**) VSNR-HardTh; (**g**) VSNR-TV; (**h**) VSNR-BiShrink; (**i**) VSNR-SLD; (**j**) WFAF; and (**k**) the proposed method.

**Figure 16:**
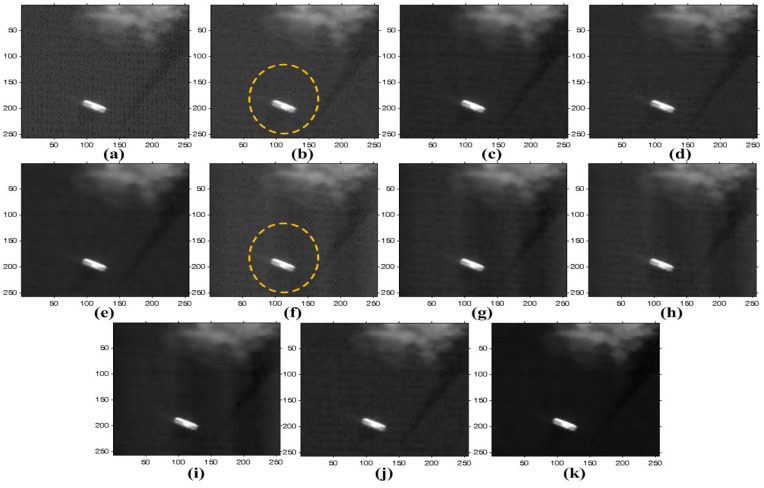
Results of various enhancement methods using the KOMPSAT-3 image of Figure 10b: (**a**) original image; (**b**) WFFT-HardTh; (**c**) WFFT-TV; (**d**) WFFT-BiShrink; (**e**) WFFT-SLD; (**f**) VSNR-HardTh; (**g**) VSNR-TV; (**h**) VSNR-BiShrink; (**i**) VSNR-SLD; (**j**) WFAF; and (**k**) the proposed method.

**Figure 17:**
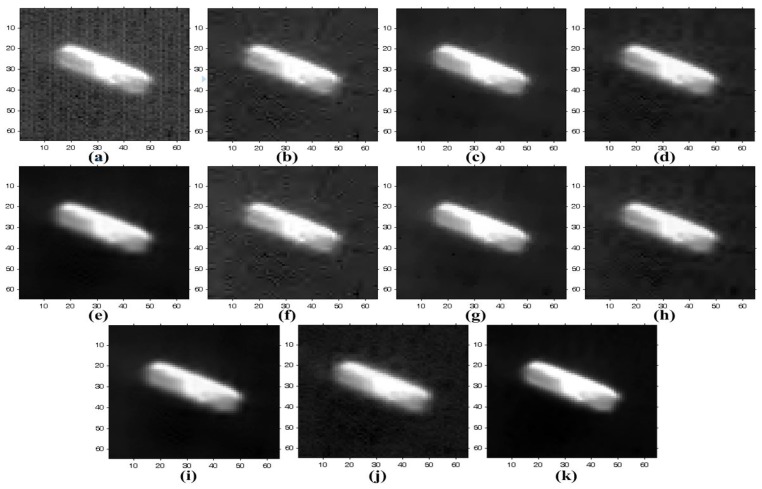
Enlarged version of cropped regions of images from Figure 16: (**a**) original image; (**b**) WFFT-HardTh; (**c**) WFFT-TV; (**d**) WFFT-BiShrink; (**e**) WFFT-SLD; (**f**) VSNR-HardTh; (**g**) VSNR-TV; (**h**) VSNR-BiShrink; (**i**) VSNR-SLD; (**j**) WFAF; and (**k**) the proposed method.

The proposed method is also tested to enhance real noisy images acquired by KOMPSAT-3, which has a push-broom-type image sensor to acquire 0.7-meter ground sample distance (GSD) panchromatic and 2.8-m GSD multi-spectral images. KOMPSAT-3 was launched into a Sun synchronous low Earth orbit in 2012. Three test images are shown in Figure 10, where there are non-periodic stripes and a certain amount of random noise. Figure 15, Figure 16 and Figure 17 respectively show the enhanced versions of Figure 10a,b using different denoising methods.

**Table 1:** PSNR comparisons of different enhancement methods for the simulated QuickBird-2 image in the panchromatic band.

Image	Degraded	WFFT−HardTh	WFFT−TV	WFFT−BiShrink	WFFT−SLD	VSNR−HardTh	VSNR−TV	VSNR−BiShrink	VSNR−SLD	WFAF	Proposed
Figure 9a	18.82	22.42	24.29	23.02	23.74	22.43	24.32	22.88	23.54	21.56	**24.71**
Figure 9b	19.30	23.74	25.59	25.22	25.83	23.53	25.35	25.13	23.36	22.92	**25.97**
Figure 9c	19.15	23.61	25.49	25.16	25.13	23.20	24.87	24.49	24.71	22.77	**26.15**
Average	19.09	23.25	25.12	24.46	24.90	23.05	24.84	24.16	24.53	22.41	**25.61**

**Table 2:** SSIM comparisons of different enhancement methods for the simulated QuickBird-2 image in the panchromatic band.

Image	Degraded	WFFT−HardTh	WFFT−TV	WFFT−BiShrink	WFFT−SLD	VSNR−HardTh	VSNR−TV	VSNR−BiShrink	VSNR−SLD	WFAF	Proposed
Figure 9a	0.458	0.662	0.767	0.764	0.780	0.658	0.763	0.760	0.771	0.620	**0.793**
Figure 9b	0.350	0.654	0.749	0.753	0.784	0.668	0.756	0.760	0.785	0.585	**0.796**
Figure 9c	0.496	0.619	0.700	0.695	0.697	0.617	**0.702**	0.697	0.692	0.575	0.696
Average	0.434	0.645	0.738	0.737	0.753	0.647	0.740	0.739	0.749	0.593	**0.761**

**Table 3:** UIQI comparisons of different enhancement methods for the simulated QuickBird-2 image in the panchromatic band.

Image	Degraded	WFFT−HardTh	WFFT−TV	WFFT−BiShrink	WFFT−SLD	VSNR−HardTh	VSNR−TV	VSNR−BiShrink	VSNR−SLD	WFAF	Proposed
Figure 9a	0.496	0.619	0.700	0.695	0.697	0.617	**0.702**	0.697	0.692	0.575	0.696
Figure 9b	0.341	0.431	0.501	0.485	0.498	0.436	0.509	0.491	0.508	0.420	**0.515**
Figure 9c	0.429	0.565	**0.643**	0.634	0.638	0.557	0.636	0.630	0.632	0.530	0.636
Average	0.422	0.538	0.614	0.604	0.611	0.536	0.615	0.606	0.610	0.508	**0.615**

As shown in Figure 15f–i, the VSNR destriping method broke the regular pattern of stripes. As a result, the subsequent denoising results exhibit a large amount of residual at the low-frequency region. In Figure 15e,i, the stripe noise is successfully removed, and the random noise is then removed by WFFT-SLD and VSNR-SLD. On the other hand, however, the SLD method generates blurring artifacts at the high-frequency regions. In Figure 15b–d,j, the stripe noise is removed successfully, while the random noise smoothing process generates the Gibbs-like artifacts because of the loss of high-frequency components in the wavelet domain.

As shown in Figure 16b,f, the HardTh denoising process produces Gibbs-like artifacts in the low-frequency region. Figure 16c,d,g,h,j show undesired artifacts near the edge and in flat regions. Although Figure 16e,i provides the better denoising results, it cannot successfully preserve the high-frequency details. As shown in Figure 15, Figure 16 and Figure 17, the proposed method gives the best denoising results in the sense of the subjective measure.

**Figure 18:**
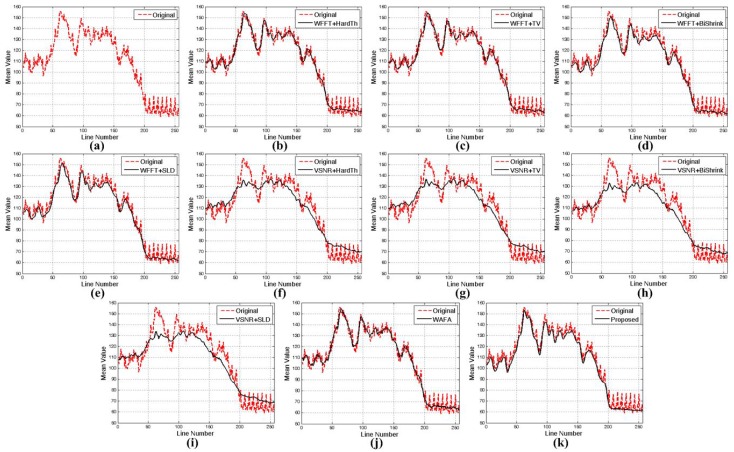
Mean cross-track profiles of the images in Figure 15: (**a**) original image; (**b**) WFFT-HardTh; (**c**) WFFT-TV; (**d**) WFFT-BiShrink; (**e**) WFFT-SLD; (**f**) VSNR-HardTh; (**g**) VSNR-TV; (**h**) VSNR-BiShrink; (**i**) VSNR-SLD; (**j**) WFAF; and (**k**) the proposed method.

**Figure 19:**
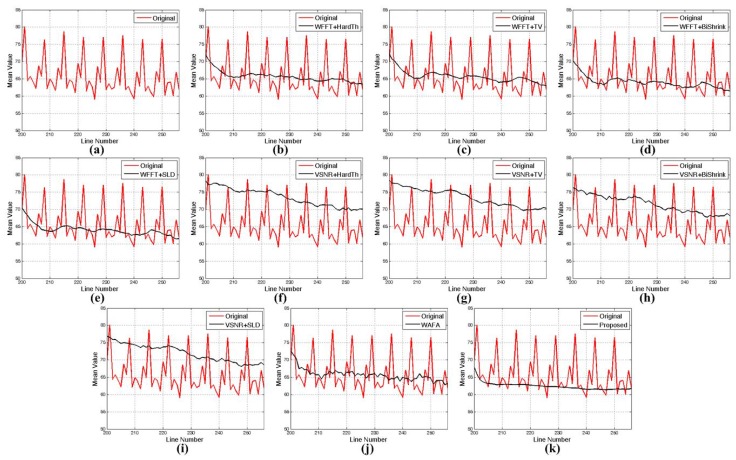
Detailed regions cropped from Figure 18: (**a**) original image; (**b**) WFFT-HardTh; (**c**) WFFT-TV; (**d**) WFFT-BiShrink; (**e**) WFFT-SLD; (**f**) VSNR-HardTh; (**g**) VSNR-TV; (**h**) VSNR-BiShrink; (**i**) VSNR-SLD; (**j**) WFAF; and (**k**) the proposed method.

Figure 18 and Figure 19 show the mean cross-track profiles, where the proposed method gives a smoothed version of the most similar curve to that of the original image.

In this work, NR [13] values are obtained as:

(17) $$NR=\frac{N_{o}}{N_{d}}$$

where $N_{o}$ represents the power of the frequency signals produced by stripe noise in the input image and $N_{d}$ represents the power of the frequency signals produced by destriped signal in the restored image.

ICV [22] is obtained for two homogeneous region of a size of 10 × 10 within the restored image as:

(18) $$ICV=\frac{N_{a}}{N_{sd}}$$

where $N_{a}$ represents the response of the homogeneous region and is obtained by averaging the pixels within the window and $N_{sd}$ represents the noise components estimated by the estimated standard deviation of the pixel within the window.

The Q-metric, NR and ICV values of KOMPSAT-3 images shown in Figure 10 are computed using ten different denoising methods, as summarized in Table 4, Table 5 and Table 6.

**Table 4:** Metric Q comparisons of different enhancement methods for the KOMPSAT-3 image in the panchromatic band.

Image	Original	WFFT−HardTh	WFFT−TV	WFFT−BiShrink	WFFT−SLD	VSNR−HardTh	VSNR−TV	VSNR−BiShrink	VSNR−SLD	WFAF	Proposed
Figure 10a	20.56	23.14	22.59	22.21	22.33	22.70	22.31	21.74	21.87	21.10	**25.04**
Figure 10b	1.75	3.63	3.81	3.69	3.40	3.60	**3.85**	3.72	3.47	2.55	3.63
Figure 10c	18.92	21.40	20.88	21.70	20.52	21.15	20.40	21.18	19.94	19.46	**23.18**
Average	13.74	16.05	15.76	15.86	15.41	15.81	15.52	15.54	15.09	14.37	**17.28**

**Table 5:** Noise reduction ratio (NR) comparisons of different enhancement methods for the KOMPSAT-3 image in the panchromatic band.

Image	Original	WFFT−HardTh	WFFT−TV	WFFT−BiShrink	WFFT−SLD	VSNR−HardTh	VSNR−TV	VSNR−BiShrink	VSNR−SLD	WFAF	Proposed
Figure 10a	1	1.7853	1.9489	1.4021	1.4814	1.7765	1.9366	1.4003	1.4696	1.7690	**2.4147**
Figure 10b	1	2.0539	5.45	4.0087	5.7267	2.0962	5.4157	4.004	5.7296	2.6924	**6.9152**
Figure 10c	1	2.2573	2.6051	2.4715	1.6835	2.2593	2.6139	2.4816	1.6897	2.2068	**3.2479**

**Table 6:** Inverse coefficient of variation (ICV) comparisons of different enhancement methods for the KOMPSAT-3 image in the panchromatic band.

Image	Sample	Original	WFFT−HardTh	WFFT−TV	WFFT−BiShrink	WFFT−SLD	VSNR−HardTh	VSNR−TV	VSNR−BiShrink	VSNR−SLD	WFAF	Proposed
Figure 10a	Sample1	11.3914	8.0220	13.8658	13.3367	11.6955	9.3089	14.2538	13.2787	11.3914	6.4331	**16.5147**
Figure 10a	Sample2	7.0652	7.1204	6.9086	5.3909	6.8935	6.9596	6.85	7.20	5.3852	7.0652	**7.6528**
Figure 10b	Sample1	11.2930	3.5298	7.9367	6.8015	6.5671	9.3363	8.5845	6.8438	11.2930	12.6230	**20.8445**
Figure 10b	Sample2	9.3522	16.6750	12.6327	6.4791	7.8812	16.5042	15.5225	5.9074	9.3522	7.9397	**20.5279**
Figure 10c	Sample1	21.5110	12.9615	21.7596	31.6765	17.7620	13.0967	24.2668	34.7859	21.5110	12.8021	**51.4480**
Figure 10c	Sample2	3.3801	3.9053	3.5540	2.6238	4.0188	3.7778	3.2521	2.4018	3.3801	3.8633	**3.9357**

### 4.3. Applications

In this subsection, the proposed method is applied to the pan-sharpening (image fusion) process, which is tested on real noisy panchromatic and multispectral images acquired by KOMPSAT-3. The goal of these methods is to improve the spatial resolution of low-resolution (LR) multispectral images using the detail of the corresponding high-resolution (HR) panchromatic image. In order to generate the VHR satellite color image, an image fusion method, such as intensity-hue-saturation (IHS) [40], is widely used in remote sensing fields. Although the pan-sharpening result is degraded if the multispectral and panchromatic images contain the stripe and random noise, the proposed method can significantly improve the quality of pan-sharpened image without undesired artifacts.

**Figure 20:**
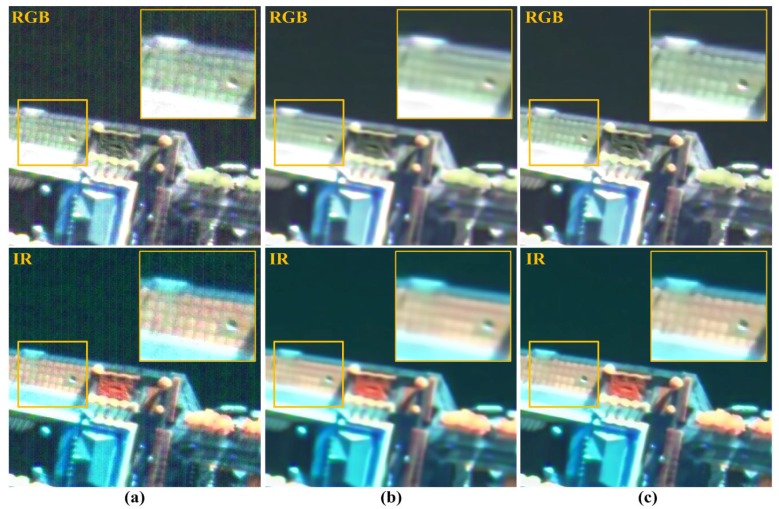
Pan-sharpening results using: (**a**) original noisy images; (**b**) block matching and three-dimensional (BM3D); and (**c**) the proposed method.

For the experiment, proposed combined wavelet-Fourier filter are preformed to remove the stripe noise, and random noise is then removed using the proposed multiscale NLM filter and BM3D [10] denoising filter, respectively. Finally, a noise-free VHR satellite color image is obtained using the IHS image fusion process from the filtered panchromatic and multispectral images. Figure 20 shows image fusion results for the real KOMPSAT-3 satellite image. As shown in Figure 20b,c, the proposed and BM3D methods successfully removed both stripe and random noise. Specifically, the BM3D filter lost the details of signal and generated blurring artifacts. On the other hand, the proposed method removed noise without blurring artifacts.

## 5. Conclusions

In this paper, a combined destriping and denoising method is presented to enhance the very high-resolution (VHR) push-broom-type satellite data. In order to preserve the radiometric integrity of satellite data, the proposed method first performs the combined wavelet-Fourier filtering to separate the stripe noise from mixed noise in wavelet LL and HL sub-bands using NVF data. Next, a modified NLM filter removes the random noise in all wavelet sub-bands. More specifically, since the push-broom-type image sensor is assumed to generate a unidirectional stripe noise pattern, the proposed method can remove the stripe noise without affecting the statistical distribution of random noise in the satellite data. Therefore, the proposed method can selectively remove stripe and random noises in the wavelet domain.

Experimental results show that the proposed method can successfully remove both stripe and random noise and preserve the geometrical structures and details of the image. Both qualitative and quantitative assessments demonstrate that the proposed method better removes the noises in real satellite images acquired by the VHR push-broom-type sensor than existing methods in term of objective and subjective measures. In addition, the proposed method can be applied to the pan-sharpening process and provides the high-quality satellite color images from the input noisy image data.
